# Investigation of an Increased Particle Size Distribution of Ti-6Al-4V Powders Used for Laser-Based Powder Bed Fusion of Metals

**DOI:** 10.3390/ma17122942

**Published:** 2024-06-15

**Authors:** Ina Ludwig, Maximilian Kluge

**Affiliations:** Fraunhofer Research Institution for Additive Manufacturing Technologies IAPT, 21029 Hamburg, Germany; maximilian.kluge@iapt.fraunhofer.de

**Keywords:** PBF-LB/M, Ti-6Al-4V, metal powder, powder blending, increased PSDs, flowability, cost reduction

## Abstract

This study investigates the potential benefits of integrating coarser particle size distributions (PSDs) of 45–106 µm into laser-based powder bed fusion of metals (PBF-LB/M), aiming to reduce costs while maintaining quality standards. Despite the considerable advantages of PBF-LB/M for producing intricate geometries with high precision, the high cost of metal powders remains a barrier to its widespread adoption. By exploring the use of coarser PSDs, particularly from electron beam-based powder bed fusion of metals (PBF-EB/M), significant cost-saving opportunities are identified. Through a comprehensive powder characterization, process analysis, and mechanical property evaluation, this study demonstrates that PBF-LB/M can effectively utilize coarser powders while achieving comparable mechanical properties as those produced with a 20–53 µm PSD. Adaptations to the process parameters enable the successful processing of coarser powders, maintaining high relative density components with minimal porosity. Additionally, market surveys reveal substantial cost differentials between PBF-LB/M and PBF-EB/M powders, indicating a 40% cost reduction potential for the feedstock material by integrating coarser PSDs into PBF-LB/M. Overall, this study provides valuable insights into the economic and technical feasibility of printing with coarser powders in PBF-LB/M, offering promising avenues for cost reduction without compromising quality, thus enhancing competitiveness and the adoption of the technology in manufacturing applications.

## 1. Introduction

Additive manufacturing (AM), particularly laser-based powder bed fusion of metals (PBF-LB/M), has emerged as a transformative technology with profound implications for various industries. PBF-LB/M offers unparalleled capabilities for producing complex geometries with high precision and reduced material waste, disrupting traditional manufacturing processes. Its ability to fabricate components with intricate designs, previously unattainable through conventional methods, has positioned PBF-LB/M as a cornerstone technology in the era of Industry 4.0.

Despite its remarkable advantages, the widespread adoption of PBF-LB/M still faces challenges, particularly concerning costs. The cost of metal powders as a feedstock material remains a critical barrier to the broader application of PBF-LB/M [1]. To address this issue, exploring strategies to reduce manufacturing costs without compromising quality has become imperative [2,3]. To achieve optimal printing outcomes, PBF-LB/M has predominantly relied on particle size distributions (PSDs) of 20–53 or 20–63 µm, matching the typically used layer thicknesses of around 30–60 µm [4,5]. Abu-Lebdeh et al. have indicated that small particles play a crucial role in filling the gaps between larger particles [6]. Larger particles (>53 µm) alone lead to a reduction in both apparent and tapped densities. In contrast, smaller particles exhibit higher interparticle forces, causing them to agglomerate and, consequently, decrease flowability, as reported by Li et al., Baitimerov et al., and Sun et al. [1,7,8]. This diminished flowability can result in irregularities within the powder bed, negatively impacting the relative density, mechanical properties, and accuracy, and increasing surface roughness. Conversely, larger particles enhance flowability, as measured by Hall flowability, avalanche angles, and cohesion indices [5,9]. Thus, the characteristics of the metal powder significantly influence flowability, their application on the build plate and, subsequently, the mechanical properties of the printed part.

One promising approach to cost reduction lies in the utilization of coarser PSDs, up to 100 µm, in PBF-LB/M [10,11]. Alfaify et al. demonstrated the processability of 20–50, 45–80, and 50–105 µm powders, using a 200 W fiber laser with a pulsed laser wave [12]. Xue et al. investigated the tensile properties of a wide range of PSDs, of 15–75 and 15–105 µm, using a 400 W ytterbium fiber laser. Applying the same process parameters as those for PBF-LB/M PSDs of 15–50 µm led to a slight decrease in strength [13]. This study aims to explore the feasibility and potential benefits of integrating coarser PSDs of 45–106 µm into PBF-LB/M. Additionally, a wider PSD of 20–100 µm is investigated with a one-on-one blend of PBF-LB/M_20-53 and PBF-EB/M_45-106 powders. The simulation results of Akib et al. demonstrated increased flowability with a mix of small and large particles [14]. This approach is validated with a wide distribution of 20–100 µm. 

The influences of the increased PBF-EB/M_45-106 and blended Ti64_20-100 powders on the powder characteristics are investigated to understand how using these coarser PSDs impacts morphology and flowability, and to compare them with the reference powder PBF-LB/M. The aim of this investigation is to identify potential improvements in powder handling and processing that could lead to more efficient and cost-effective production. A process parameter study is performed for each powder to identify the process window for dense samples (>99.9%). Mechanical specimens are printed with customized process parameters to assess their manufacturability into defect-free parts that meet industry standards for relative density, microstructure, hardness, and tensile properties. 

Through a comprehensive market survey and analysis of the cost reduction potentials of the feedstock material, we investigate how the adoption of PBF-EB/M powders can contribute to lowering manufacturing costs compared to commonly used and more expensive powder blends with PSDs of approximately 20–53 µm while maintaining comparable part quality. By shedding light on the economic and technical viability of printing with PBF-EB/M PSDs, this study aims to provide valuable insights for industry stakeholders and researchers alike. By using laser powers around 400 W, as well as giving clear price and cost indications, this study provides a novel approach to this research topic. Ultimately, the findings aim to facilitate the advancement and widespread adoption of PBF-LB/M technology by addressing critical cost-related challenges and enhancing its competitiveness within the manufacturing landscape.

## 2. Materials and Methods

### 2.1. Powder Characterization

**Figure 1:**
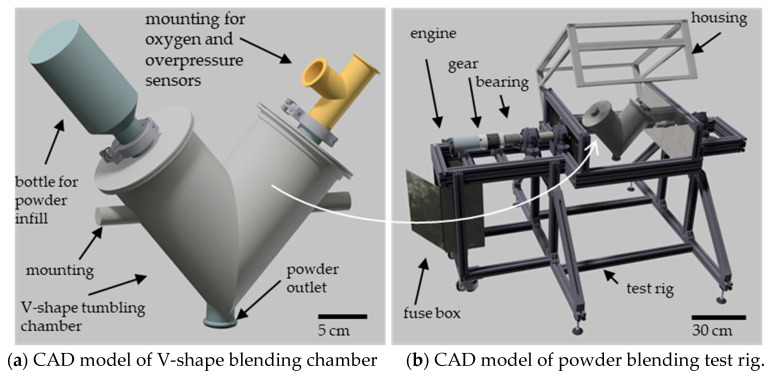
(**a**) CAD model of V-shape powder blender and (**b**) the surrounding test bench.

Two plasma-atomized Ti-6Al-4V powders of ELI quality were purchased with typical PSDs for PBF-LB/M (20–53 µm, referred to as PBF-LB/M_20-53) and PBF-EB/M (45–106 µm, referred to as PBF-EB/M_45-106). Both powders were blended in a custom 3l-volume, V-shape, laboratory-size tumbling blender, as shown in Figure 1. The powders were blended in a one-on-one ratio with a 70% filling level inside the V tumbler. The blending was performed for 15 min at 40 rpm (rounds per minute). Afterwards, the blended powder was sieved with a 100 µm mesh size to obtain a defined PSD of 20–100 µm (Ti64_20-100).

All three powders were characterized by particle shape, size, chemistry composition, and flowability. The PBF-LB/M powder served as the reference for comparing the flowability among the three powders. The characterization of particle shape included measurements of sphericity (SPHT), symmetry (Symm), and aspect ratio (w/l—width-to-length ratio). The particle size was displayed using deciles for 10, 50, and 90% (D10, D50, and D90) along with a frequency distribution curve. These morphology characteristics were measured using the dynamic image analysis of the X2 Camsizer, according to ISO 13322-2 [15]. The morphology values were determined based on the x_area_ diameter, which describes the measured area of the particle as an equivalent circle.

The chemical compositions of the base powders, PBF-LB/M and PBF-EB/M, were taken from the suppliers’ data sheets. The Ti, Al, V, and Fe were measured using Inductively Coupled Plasma Optical Emission Spectroscopy (ICP-OES) according to ASTM E2371 [16]. The O and N were determined by Inert Gas Fusion according to ASTM E1409 [17]. The H was measured with Inert Gas Fusion with detection using thermal conductivity (IGF-TC) according to ASTM E1447. The C was determined by combustion analysis (CA) according to ASTM E1941 [18,19].

**Figure 2:**
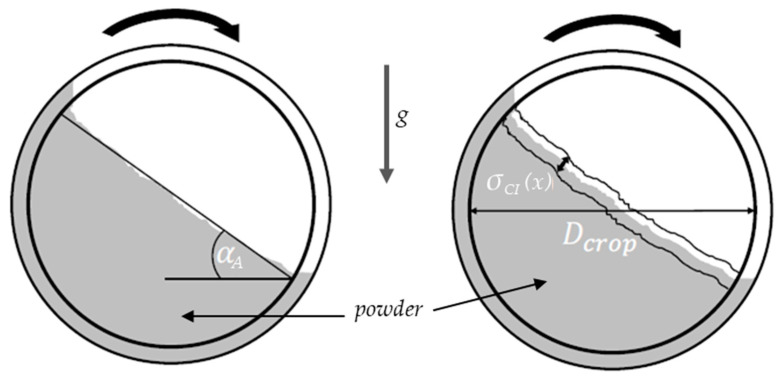
Schematic image of the GranuDrum from the front view [24].

The flowability was characterized using Hall funnel and GranuDrum tests. In accordance with DIN EN ISO 3923-1 and DIN EN ISO 3953, the apparent and tapped densities were measured and, in accordance with DIN EN ISO 4490, the Hall flowability was determined [20,21,22]. The Hausner ratio was calculated as described by Hausner in 1981, which is the ratio of apparent to tapped density [23]. The relative humidity was measured with a hygrometer (±2%) and the temperature with a thermometer (±0.2 °C) from SwitchBot to determine the absolute humidity during the Hall funnel tests. With the GranuDrum, the avalanche angle and cohesion index were determined for increasing and decreasing rotational speeds. Therefore, 50 g of powder was poured into a glass-sided drum as demonstrated in Figure 2. The drum rotated at set speeds of 2, 4, 6, 8, 10, 15, 25, 35, 45, and 55 rpm to simulate multiple stress conditions. A backlight enabled a front camera to detect the powder surface and its movement inside the drum.

The diameter was cropped to *D_crop_* to reduce the influence of particles attached to the outer edge. The powder built up on the wall until it started to slip. The steepest gradient of the buildup describes the avalanche angle *α_A_*. The cohesion index *C* was calculated based on the temporal fluctuations of the powder interface. The calculations are shown in Equations (1) and (2):

(1) $$\sigma_{CI}\left(x\right)=\sqrt{\frac{\sum_{i=1}^{N_{y}(x)}{\left(\bar{y}\left(x\right)-y_{i}\left(x\right)\right)}^{2}}{N_{y}(x)}}$$

(2) $$C=\frac{1}{D_{crop}}\int \sigma \left(x\right)dx$$

The results were similar for both increasing and decreasing rotational speeds, which is why only the values for increasing speeds are shown for clarity [25,26].

### 2.2. PBF-LB/M Process

The powders were processed using an M2 Cusing system from Concept Laser equipped with a 400 W fiber laser with a beam diameter of 100 µm. A double-rubber recoater blade was used. The chamber was flooded with Ar to create an inert gas atmosphere. The layer thickness was fixed at 60 µm without any platform heating. Thirty-six pyramid cone density cubes were placed on a 250 mm × 250 mm build plate. The process parameters, including laser power, scanning speed, and hatch distance were varied within a fractional factorial design of experiments (DOEs). The process parameter leading to the highest relative density, a minimum of 99.9%, was chosen to print further mechanical specimens. The final volume energy density (VED) and build rate are presented in Section 3.2.

Powder samples were collected from the supply, the build plate (after build job completion), and the overflow to analyze the particle sizes. The samples were measured with dynamic image analysis, as described in Section 2.1.

### 2.3. Mechanical Properties

All the specimens used for the mechanical property analysis were still attached to the build plate while being heat-treated for 2 h at 800 °C in a vacuum for annealing to decompose α’ → α + β. They were separated afterwards via wire eroding [27].

For the warm embedding of the density cubes with the CitroPress, the polymer matrix ClaroFast was used. First, the samples were ground increasingly finer and then polished with OP-U (0.04 µm grain size). Images were taken with a VHX-5000 (manufactured by Keyence, Osaka, Japan) reflected-light microscope at 50× magnification in a dark-field exposure. Image analysis software was used to determine the relative density, similar to a greyscale analysis to detect the pores. The average relative density was calculated for the accompanying density cubes of the tensile specimens.

The microstructure was analyzed using a JEOL 7200 F (manufactured by JEOL Ldt., Tokyo, Japan) scanning electron microscope (SEM). The specimens were connected with a copper band to the socket to ensure sufficient conductivity. The images were taken in backscattered electron (BSE) mode.

**Figure 3:**
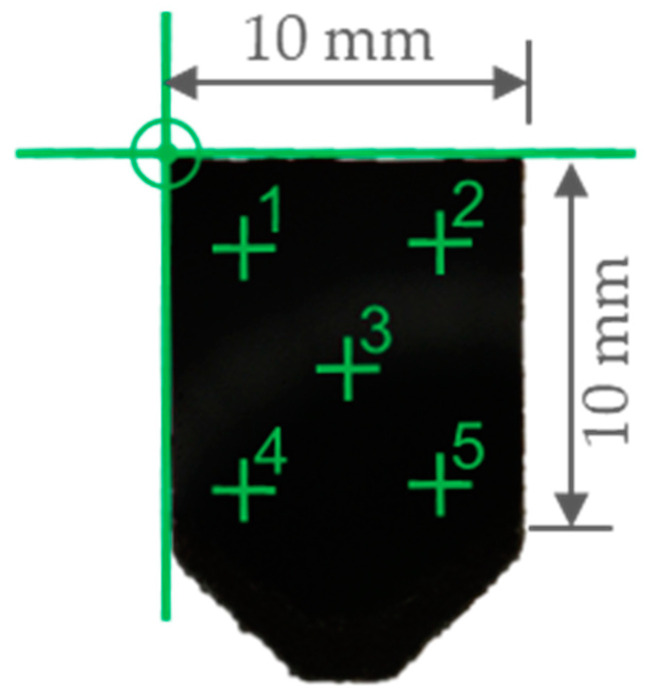
Schematic image of a longitudinal section of a density cube with the used imprint pattern for HV10 measurements. The crosshair (incl. circle) indicates the orientation of the measuring points number 1–5 marked with +.

The hardness was measured on the polished surfaces of the density cubes. In according with ISO 6507-1, the Vickers hardness HV10 was determined using a DuraScan (manufactured by Struers GmbH, Willich, Germany) [28]. At five points per density cube, imprints were made, as shown in Figure 3. The mean values are displayed in a bar chart, including the standard deviation. The results are compared to industry standards, which are highlighted with a green bar.

The tensile properties—tensile strength (*R_m_*), yield strength at 0.2% (*R*_p0.2_), elongation at break (*A*), and reduction in area (*Z*)—were characterized using horizontally and vertically oriented tensile specimens in Form B, according to DIN 50125, with a 5 mm diameter [29]. An offset of 1 mm was machined to eliminate any influence of the as-built surface roughness. At 20 °C, the tensile tests were performed according to DIN EN ISO 6892-1, using a Galdabini-Quasar 600 kN/50 kN (manufactured by Galdabini S.p.A., Cardano al Campo, Italy) [30].

The correlation analysis was conducted using the statistical software R (RStudio 2023.06.0 Build 421) based on Pearson correlation tests. Pearson correlation tests assess the linear correlations between two variables. A correlation was considered statistically significant if the Pearson correlation coefficient (r_C_) was ≥0.7, with a significance level (α_C_) of ≥5%. Such correlations are classified as high in the literature, ensuring robust statistical relevance. The causal relationship was examined for each validated correlation. The results are visualized in a correlation matrix, where blue indicates a positive correlation and red indicates a negative correlation. The size of the circle corresponds to the significance level, with larger circles representing higher significance [31].

The mechanical properties of the investigated powders were compared to industry standards, and are presented in Table 1.

### 2.4. Economics

A market survey was undertaken to assess the potential cost savings achievable through the utilization of increased PSDs of 20–100 µm, with a blend of PBF-LB/M and PBF-EB/M powders. Offers were solicited from 22 powder suppliers, including both traders and atomizers, for Ti-6Al-4V ELI grade 23 in quantities of 100 kg and 1000 kg, specifically in PBF-EB/M and PBF-LB/M PSDs. Machine suppliers were not included in this survey. The prices were not negotiated, and all offers were collected between 1 June and 31 August 2021. Currency conversions were performed on 2 September 2021, to standardize the prices. After identifying four outliers, the analysis was based on a database of eleven suppliers. The offers for PBF-LB/M powders were summarized to cover typical PSDs ranging between 20–53 µm and 20–63 µm. Similarly, for the PBF-EB/M powders, the PSDs considered were summarized to include 44–106 µm, 45–100 µm, and 45–125 µm. It is worth noting that suppliers may have different standards for the PSDs they can offer, making it difficult to compare the same PSDs across suppliers.

## 3. Results

### 3.1. Powder Characterization

In Table 2, the results of the powder morphology are presented. The particle shapes are similar, with deviations between the three powders of 0.008 for sphericity, 0.028 for symmetry, and 0.037 for the aspect ratio. The decile values of PBF-LB/M_20-53 and PBF-EB/M_45-106 are within an approximately 10% deviation from the PSDs stated in the suppliers’ powder certificates. Ti64_20-100 has a D90 value of 80.7 µm, with a deviation of around 20%.

**Figure 4:**
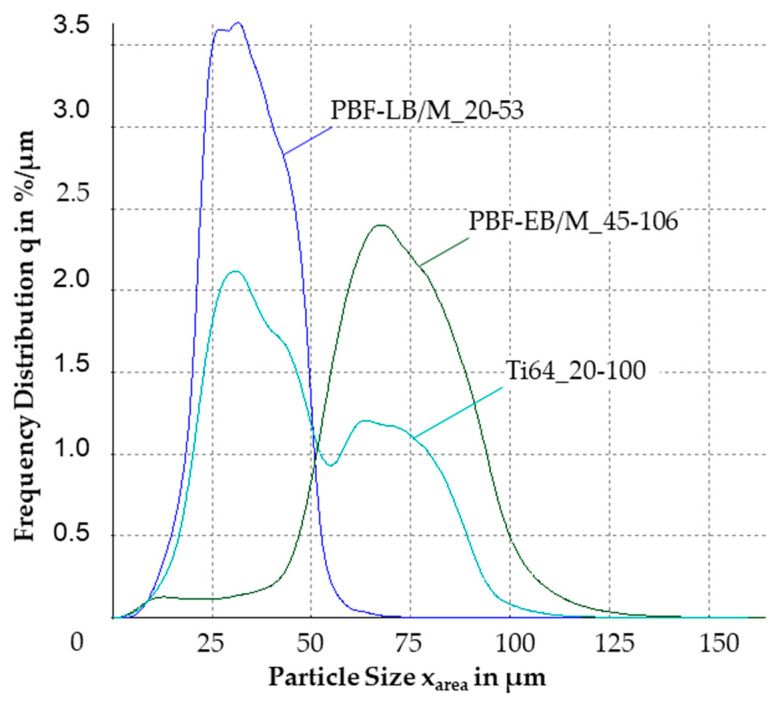
Diagram of the frequency distributions of the investigated powders.

The frequency distribution is exhibited in Figure 4. Both PBF-LB/M_20-53 and PBF-EB/M_45-106 show a Gaussian-shape distribution. Ti64_20-100 has a bimodular distribution, with two local maxima around the modal values of the other two powders.

In Table 3, the chemical compositions of the PBF-LB/M and PBF-EB/M powders are compared to the ELI standard. No relevant influence of the blending procedure on the chemical composition was expected; therefore, Ti64_20-100 was not analyzed.

The flowability, measured with the Hall funnel, is exhibited for all three powders in Table 4. PBF-EB/M demonstrates a lower apparent and tapped density than the other two powders. There is a small deviation in the Hausner ratios, with a maximum difference of 0.04 between PBF-LB/M_20-53 and Ti64_20-100. The Hall flowability of PBF-EB/M_45-106 is higher than that of the other two powders at 25.2 s/50 g, although PBF-LB/M_20-53 was not free-flowing and additional tapping was required. The relative humidity was measured on each test day and converted into the absolute humidity.

**Figure 5:**
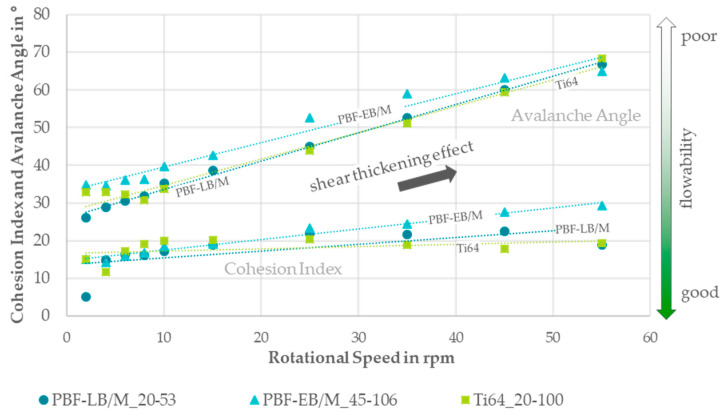
Diagram of the cohesion indices and avalanche angles over the set rotational speed range for all three investigated powders.

In Figure 5, the avalanche angles and cohesion indices are presented for increasing rotational speeds for all three powders. The curve progression increases similarly for all the powders. The PBF-EB/M_45-106 powder exhibits higher values compared to the other two powders, which have nearly identical values.

### 3.2. PBF-LB/M Process

Table 5 provides an overview of the variation range per parameter and the number of build jobs required to achieve the desired 99.9% relative density.

The two bottom lines present the volume energy and build rate of the final process parameters used for the mechanical specimen build job. The final process parameters for PBF-EB/M_45-106 and Ti64_20-100 were identical, hence the similarity in VED and build rate. These process parameters differed from those of the PBF-LB/M_20-53 process parameters, with a hatch distance 0.05 mm smaller, a laser power 10 W higher, and scanning speed 300 mm/s slower.

**Figure 6:**
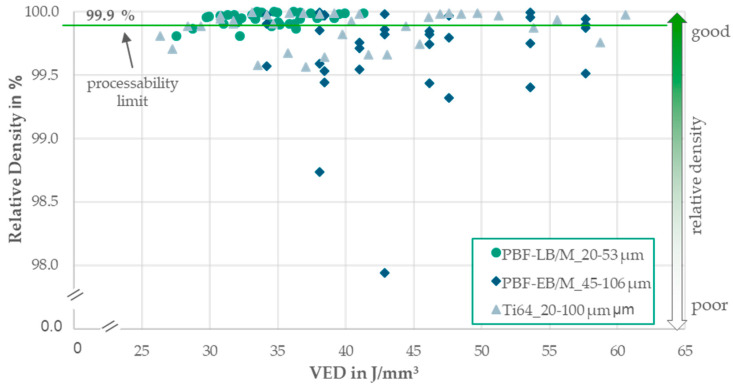
Diagram of the relative density per volume energy for each PBF-LB/M parameter combination per PSD variation.

Figure 6 displays the achieved densities for each process parameter combination, focusing on the relative density per volume energy. All the powders were successfully processed into cubes with densities surpassing 99.9%. The PBF-LB/M_20-53 powder yielded the highest number of density cubes exceeding 99.9%. The blended Ti64_20-100 powder demonstrated consistently high densities across a broad VED range from 30 to 60 J/mm^3^, although some values exhibited drops. While certain values of the PBF-EB/M_46-106 powder achieved high densities, no distinct process window could be identified. Processability was evident for all three powders.

**Figure 7:**
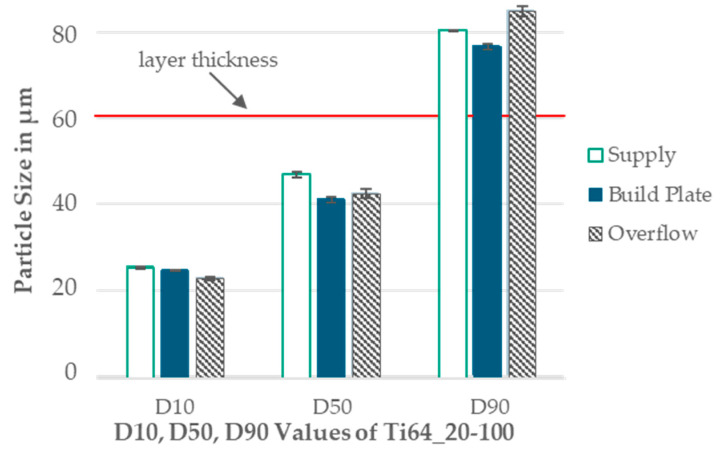
Bar chart of the particle size from the supply, build plate, and overflow measured for Ti64_20-100.

The Ti64_20-100 powder’s application was tested to encompass the entire investigated PSD range of 20–100 µm. The results are presented in a column diagram in Figure 7. A slight decrease of approximately 1–5 µm is observed for the samples collected at the supply to those collected at the build plate for D10, D50, and D90. The samples from the overflow exhibit a coarsening of 1 µm for D50 compared to those at the build plate. For D90, there is a coarsening of 5 µm compared to the supply samples, and 8 µm compared to the samples taken from the build plate. D10 continues to decline in the overflow.

### 3.3. Mechanical Properties

**Figure 8:**
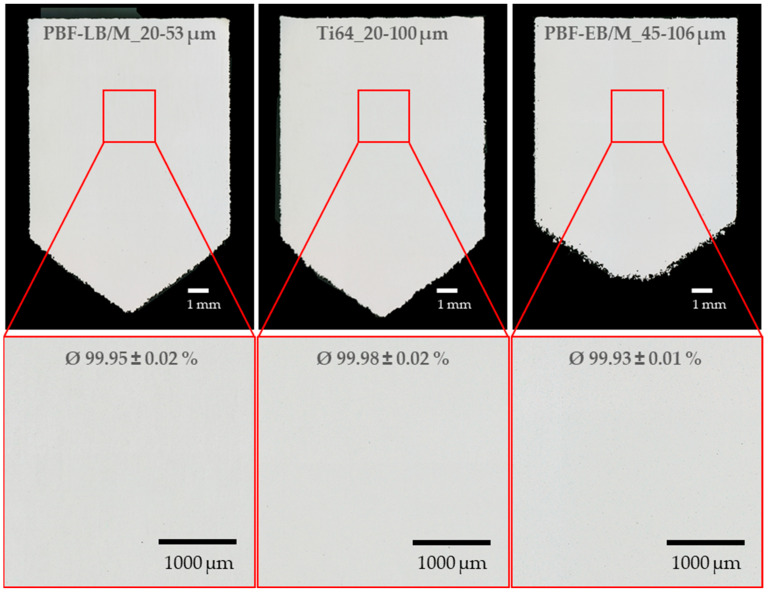
Light microscope images of polished density cubes for relative density analysis.

All three powders were successfully processed into cubes with densities exceeding 99.9%, demonstrating a low standard deviation, with a maximum of 0.02%. Minimal pores were detected, as illustrated by the exemplary specimens of each powder in Figure 8.

**Figure 9:**
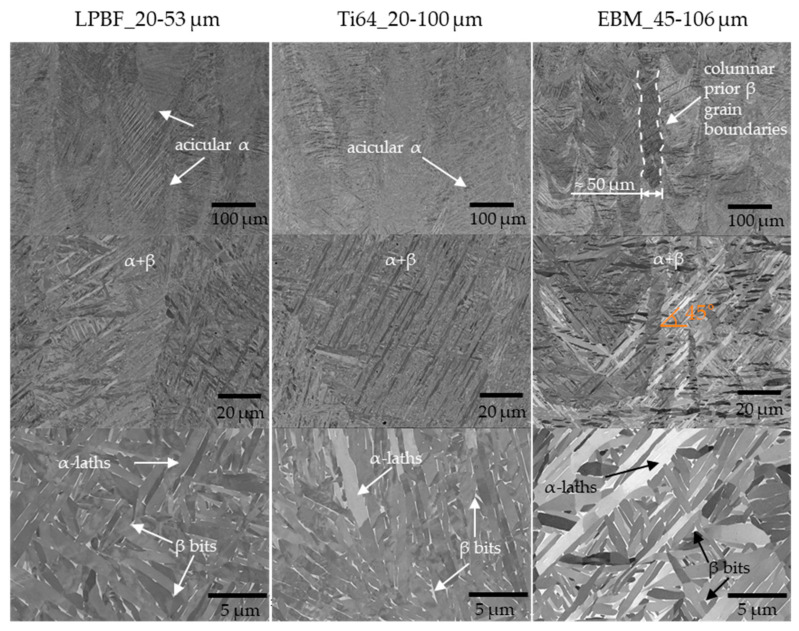
Exemplary SEM images for each investigated powder for microstructural analysis.

The microstructures of the specimens were analyzed exemplarily, as shown in Figure 9. The microstructures reveal similar columnar prior, β-grain boundaries partially filled with acicular α-structures. White β-bits are identified. There are no discernable differences in the microstructure of the three powders attributable to the two different process parameters.

**Figure 10:**
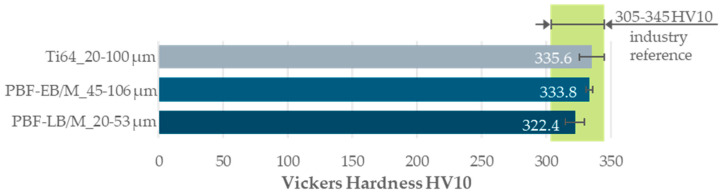
Bar chart of the measured Vickers hardness HV10 for each investigated powder compared to the industry references.

For all three powders, Vickers hardness within the industry standards range of 305–345 HV10 was achieved. The industry standard is indicated by the green bar in Figure 10. The PBF-LB/M_20-53 powder exhibits the lowest hardness at 322.4 HV10, while the wide distribution of Ti64_20-100 displays the highest standard deviation of 9.7 HV10.

**Figure 11:**
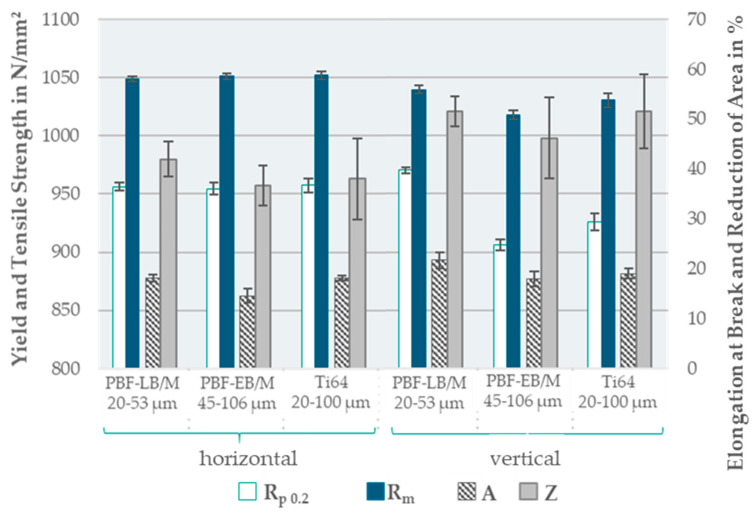
Diagram of the measured tensile properties in a horizontal and vertical orientation for each powder after heat treatment (800 °C for 2 h).

The results of the tensile tests are illustrated in Figure 11. All three PSDs achieved target tensile properties within the industry standard values that are presented in Table 1.

In the horizontal orientation, the tensile strength and yield strength results were similar for all three powders. Both PBF-LB/M_20-53 and Ti64_20-100 exhibited a similar elongation, which was four percentage points higher than that of the PBF-EB/M_45-106 powder. The reduction in area of PBF-EB/M_45-106 was close to the results of Ti64_20-100, with both achieving around a 4–6% lower reduction in area compared to PBF-LB/M_20-53.

In the vertical orientation, there was a decrease in the strength of PBF-EB/M_45-106 and Ti64_20-100 by 31 and 48 N/mm^2^. For PBF-LB/M, only the tensile strength was reduced by 10 N/mm^2^, while the yield strength was increased by 14 N/mm^2^. The elongation, as well as the reduction in area, were enhanced for all three powders by around 10%, reaching values of over 20% for PBF-LB/M_20-53. In both orientations, the standard deviation for all reduction in area results was the highest, reaching up to 8% (PBF-EB/M—vertical and Ti64_20-100—both orientations).

**Figure 12:**
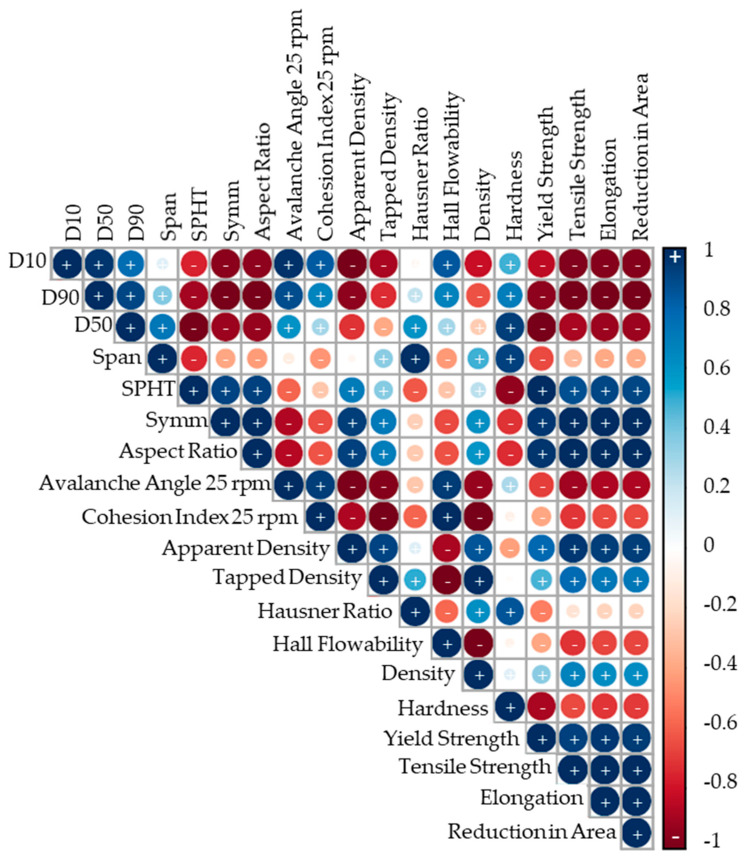
Correlation matrix.

The correlation analysis is visualized in an upper-half correlation matrix, shown in Figure 12. Positive correlations are observed between the D10, D50, and D90 deciles and the avalanche angles and cohesion indices, while negative correlations are noted for the apparent and tapped densities, as well as for the tensile properties. A more regular particle shape appears to reduce the avalanche angle and cohesion index while increasing the apparent and tapped densities, along with the tensile properties. Higher avalanche angles and cohesion indices correspond to a decreasing relative density and tend to reduce the tensile properties. Increased density correlates positively with the tensile properties, in contrast to hardness, which exhibits a negative correlation with the tensile properties.

### 3.4. Economics

**Figure 13:**
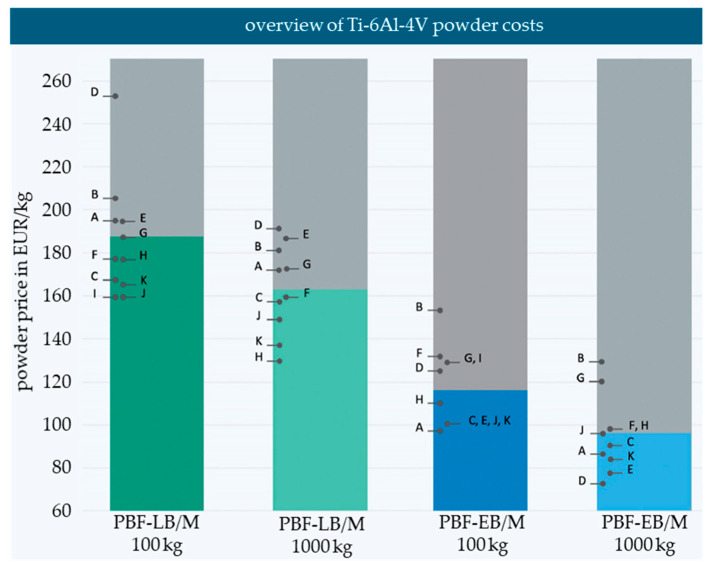
Diagram of the Ti-6Al-4V market price survey of typical PBF-LB/M and PBF-EB/M PSDs; A–K: different suppliers.

To evaluate the potential cost savings for PBF-LB/M with the additional utilization of coarser powders, such as 45–106 µm PBF-EB/M powder, a market survey was conducted. The results are displayed in Figure 13, with the individual results anonymized.

The survey revealed that, on average, the PBF-EB/M powder was 38% cheaper for a purchase quantity of 100 kg compared to the PBF-LB/M powder. For a larger purchase quantity of 1000 kg, the cost difference increased to an average of 44%.

**Figure 14:**
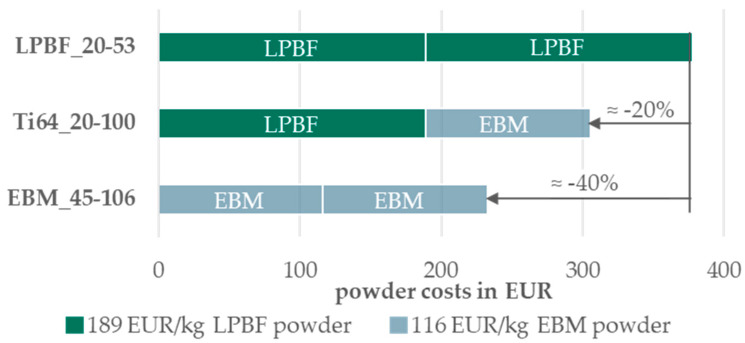
Diagram of powder costs distribution for an equal-proportion blend per investigated PSD.

The costs for the investigated powders in a one-to-one blend are illustrated in Figure 14, based on the prices for 100 kg and without additional costs calculated for the blending procedure itself. Incorporating a blend consisting of 50% PBF-LB/M powder and 50% PBF-EB/M powder to achieve a PSD of 20–100 µm results in an average cost reduction of around 20%.

Studies by Anderson and Terpstra, Qi et al., Kohlwes et al., and Li et al. have demonstrated a potential increase in atomization yield of 10–30% by increasing the PSD up to 100 µm [9,35,36].

## 4. Discussion

### 4.1. Powder Characterization

The difference in the particle shapes of PBF-LB/M and PBF-EB/M was negligible. Both could be considered spherical, with a sphericity of almost 0.9 mean SPHT, which is typical for plasma-atomized powders [37,38]. The blended powder presented a morphology that fell between the two base powders. Therefore, the influence of the blending procedure could be excluded. The D90 value of 80.7 µm for the Ti64_20-100 blend was smaller than the expected 92.1 µm for the PBF-EB/M_45-106 powder, suggesting the potential degradation of the PBF-EB/M_45-106 powder beforehand. Sieving with a 100 µm mesh size likely contributed to reducing the D90 value below 100 µm, due to irregular-shaped particles that did not pass through the mesh. However, the loss of particles between 80 and 90 µm due to powder handling was unlikely, as this is more commonly associated with particles smaller than 20 µm, owing to higher interparticle forces, such as van der Waals or hydrogen bonds. The bimodal curve from the frequency distribution was almost exactly half of the combined curves for the two base powders. This indicated that a middle part around 50 µm was missing to create a Gaussian-shape distribution based on two separately purchased powders. For PBF-LB/M_20-53, the particles > 50 µm, and for PBF-EB/M, the particles < 50 µm, were removed for sale.

Since both base powders were within the ELI limits and did not significantly differ from each other, the influence of the chemical elements on the PBF-LB/M process parameter adaptations and mechanical properties could be discounted. Contamination (abrasive wear or oxygen pick-up) from powder handling, such as blending and sieving, could be reduced for all powders by careful handling, but could not be entirely eliminated.

Flowability was assessed using the Hall funnel method. Smaller particles were observed to fill voids, resulting in higher apparent and tapped densities for PBF-LB/M_20-53 and Ti64_20-100, while PBF-EB/M_45-106 exhibited lower apparent and tapped densities. Contrary to expectations, PBF-EB/M_45-106 demonstrated a lower Hall flowability of 25.2 s/50 g. The absence of fine particles should have reduced the bonding interparticle forces to reduce the flow time. Additional tapping was needed for PBF-LB/M_20-53, explaining its lack of free-flowing behavior in the Hall funnel despite a Hall flowability of 24.67 s/50 g, making it the powder with the poorest flowability characteristics. Ti64_20-100 stood out with slightly better flowability compared to PBF-EB/M_45-106.

External factors, such as humidity, may have affected the flowability, with higher humidity potentially increasing particle cohesion. The correlation between humidity and flowability suggests that PBF-EB/M_45-106 could have exhibited similar flowability to Ti64_20-100 under similar humidity levels. However, the coarser particles in Ti64_20-100 appeared to improve flowability despite the lower humidity levels.

The flowability measurements using the rotating drum method revealed shear thickening behavior for all three powders, with Ti64_20-100 exhibiting improved flowability compared to the base powders PBF-LB/M_20-53 and PBF-EB/M_45-106. A correlation analysis confirmed lower flowability with increasing particle sizes. However, a clear trend for the correlation of particle shape and flowability was difficult to discern due to minor differences in particle shape.

### 4.2. PBF-LB/M

To explore the processability of larger PSDs of up to 106 µm for Ti-6Al-4V, the process parameters were adjusted accordingly. The process parameters for the PBF-LB/M_20-53 µm powder served as the reference for developing the process parameters for the PBF-EB/M_45-106 and Ti64_20-100 powders. The adaptation for PBF-EB/M_45-106 was based on the premise that melting the coarser particles necessitated a higher energy input, as observed in the findings of Spurek et al. and Kohlwes et al. [9,39]. For both the PBF-EB/M_45-106 and Ti64_20-100 powders, a higher VED was required to achieve relative densities comparable to those of PBF-LB/M_20-53. The reduced hatch distance for PSDs of up to 106 µm resulted in a greater overlap of scan vectors. This adjustment was effectively balanced with a lower scanning speed, which is known to stabilize melt pool dynamics and reduce spattering. However, maintaining the same scanning speed as for the PBF-LB/M_20-53 powder led to excessive energy input for coarser PSDs, resulting in increased porosity. Despite a slight increase in laser power compared to that used for PBF-LB/M_20-53, the vaporization of small particles was unlikely. Instead, the increased energy input, combined with the remelting of scan vectors due to overlap, could be seen as an in situ heat treatment for the parts. This necessitated a combination of higher scanning speed, lower hatch distance, and slightly higher laser power for the increased particle sizes to achieve relative densities comparable to those of PBF-LB/M_20-53. Further investigations are warranted to refine this approach by adapting the parameters to the PSD, potentially extending powder reuse by mitigating proven coarsening effects during reuse. Processability was achieved for all three powders with three different PSDs.

The application test (Figure 7) revealed a slight decrease in particle size in the build chamber, suggesting the potential removal of smaller particles (≤20 µm) by the gas stream during printing. The segregation of larger particles (≥75 µm) in front of the recoating blade could lead to the refinement of the PSD, as simulated by Lee et al. [40]. This segregation might have resulted in the coarser particles not being fully deposited onto the building platform, and instead being pushed into the overflow.

### 4.3. Mechanical Properties

The evaluation of the manufacturability of larger PSDs of up to 106 µm of Ti-6Al-4V primarily relied on the mechanical properties, including relative density, microstructure, hardness, and tensile properties. General values from the literature and material data sheets were used for comparison to assess manufacturability, considering varying target values based on specific applications.

All three powders consistently achieved reproducible relative densities above 99.93% and met the 99.9% target. Although a closer comparison was not meaningful due to potential nanometer-level deviations from measurement and process fluctuations, successful process parameter adaptations resulted in high relative densities for all PSDs.

The microstructure exhibited similarities among all the samples, characterized by an α-structure with β-bits resulting from the heat treatment at 800 °C for 2 h. β-columnar growth was enforced by a scanning pattern of 90° [41]. No significant influence of the PSD or other powder characteristics on the microstructure were identified, indicating that the adapted process parameters led to comparable microstructure characteristics across the different PSDs from 20–53 µm to 20–100 µm.

The Vickers hardness for all three PSDs fell within typical ranges for Ti-6Al-4V hardness achieved with PBF-LB/M. The standard deviations for Ti-6Al-4V hardness were below 3%. The variations in hardness among the PSDs were attributed to differences in cooling rates, with slower cooling rates associated with lower hardness. The adapted process parameters allowed for the achievement of typical PBF-LB/M hardness levels across all three PSDs.

The results of the tensile tests were primarily compared to industry standards. The adapted process parameters allowed for the evaluation of the manufacturability of large PSDs under melting conditions suitable for large particles. However, it reduced the direct comparability with the standard powder. The tensile specimens exhibited values comparable to industry standards, (Table 1) and therefore met the target. A slight anisotropy was detected with decreased tensile strength and increased ductility in a vertical orientation for all the PSDs. Several factors affected the mechanical properties, amongst others, the build height, PSD, and process parameters. A lower build height was less prone to irregularities and defects (6 mm in horizontal orientation compared to 51 mm in vertical orientation) as stated by Singh et al. and Xue et al. [13,42]. The findings of Yadroitsev et al. showed no influence of a heat treatment below the β-transus temperature on anisotropy [43]. Slight anisotropy persisted despite annealing at 800 °C for 2 h, suggesting a further process or heat-treatment parameter adaptation is needed. An increase in tensile properties was observed in a vertical orientation for PBF-LB/M_20-53, possibly due to the dissipated heat resulting in higher strength and ductility. The lower specific surface area of larger particles up to 106 µm varied the melting behavior compared to smaller particles of 20–53 µm, which should be compensated for with the adapted process parameters. Further adjustment of the process parameters would offer room for enhanced mechanical performance. The microstructure was analyzed using density cubes with 15 mm build height, showing no significant influence of the increased PSDs. An investigation of specimens 50 mm in height could provide more details about deviations in the microstructure that influence the mechanical properties and lead to anisotropy.

The achieved elongation at break is noteworthy for all three PSDs. PBF-EB/M_45-106, with the lowest average elongation at break of 16.3%, exceeded the industry standard value of 14.6% on average (averages values based on Table 1). The values of PBF-LB/M and Ti64_20-100 exceeded these, at 18.6 and 20%. This indicates that the used process parameters targeted ductility rather than strength.

The correlation matrix reflects the obtained results. An increased PSD decreases flowability and reduces the relative density and tensile properties. This could be because a reduced homogeneous and dense powder application, which increases the likelihood of voids and irregularities in the powder bed, influences the melt pool and, subsequently, decreases the mechanical properties. Another reason could be the adapted process parameters, which were adapted purposefully to achieve a high relative density rather than high strength or ductility. As high relative densities and industry-standard mechanical properties were achieved for all the powders, it is more likely that the process parameters affected irregularities in the powder bed. However, the complexity of the causal relationship between powder, process, and part qualities, as demonstrated by Sun et al., requires further investigations [1]. The correlation of particle shape with improved flowability and tensile properties needs to be relativized, due to the similarity of the particle shapes investigated. Studies have demonstrated the correlation of higher particle sphericity with higher mechanical properties [5]. For the Vickers hardness, it was the opposite, with higher hardness with increased PSDs and higher irregularity. The cooling rates of the different process parameters could still be the dominant factor for the hardness result. The hidden effects of the lower specific oxygen content of the coarser particles due to their lower specific surface were not considered and warrant further investigation.

Further quality criteria, such as dimensional accuracy or surface roughness, were not considered in this investigation. Agglomerated or partially melted particles of 100 µm likely increase the surface roughness compared to 53 µm particles, limiting their utilization in filigree structures. A study of the process parameters dealing with the contour parameter could address this aspect.

### 4.4. Economics

This investigation provides crucial insights for considering the purchase of additional coarser powders or switching to a broader PSD for PBF-LB/M, which is already utilized in other production processes, such as thermal spray. The analysis indicates that the price of metal powders is significantly influenced by both the order volume and the specified PSD. Notably, certain suppliers specialize in specific PSDs, which is evident in their cost structures. For example, supplier D offered the highest price (<250 EUR/kg) for PBF-LB/M powder, but at the same time offered the cheapest price (<80 EUR/kg) for PBF-EB/M powder. This suggests that market demand and scale effects play a major role in the pricing strategies of powder atomizers and suppliers.

An analysis of the purchase costs for powders up to 106 µm in particle size and larger quantities, such as 1000 kg for Ti-6Al-4V, revealed a substantial cost reduction potential of 40% for PBF-EB/M powders. This translates to a 20% reduction in feedstock powder costs when blended to a wider PSD of 20–100 µm. These cost savings align with the estimated resource savings resulting from a 10–30% higher atomization yield. However, precise calculations of the potential yield increase are challenging due to the proprietary nature of yield information from powder atomizers.

The process parameters selected for coarser PSDs resulted in lower productivity, which is reflected in the lower build rate. An overall savings through using larger PSDs is only possible with the same or higher productivity. Future studies should further investigate the influence of larger PSDs on the overall cost structure. Additionally, optimizing process parameters to enhance productivity should be analyzed to reduce overall costs.

## 5. Conclusions

In conclusion, this study provides valuable insights into the potential benefits of incorporating coarser particle size distributions up to 106 µm into laser-based powder bed fusion of metals. Through a comprehensive investigation encompassing market surveys, cost analyses, and technical evaluations, the feasibility and implications of printing with coarser powders, such as those utilized in electron-beam powder bed fusion of metals are explored.

The findings underscore the following key points:

- Cost Reduction Potential: The utilization of PSDs up to 106 µm in PBF-LB/M offers significant cost reduction opportunities for the feedstock powder, particularly when compared to the use of finer powders traditionally associated with the process, such as 20–53 µm. The market survey revealed notable cost differentials between PBF-LB/M and PBF-EB/M powders, with the latter proving to be considerably more cost-effective.
- Maintained Quality Standards: Despite the up to 40% lower cost associated with coarser powders, this study demonstrates that printing with these PSDs does not necessitate a compromise in quality. Through an analysis of the mechanical properties, microstructure, and processability, it is demonstrated that specimens manufactured using PSDs of 45–106 µm and 20–100 µm exhibit comparable mechanical performance to industry standards. In this study, deviations in elongation and in yield strength in the vertical orientation occurred for those produced with finer powders of 20–53 µm and different process parameters.
- Process Adaptability: Moreover, this research highlights the adaptability of the PBF-LB/M parameters to accommodate coarser powders. Process parameter adjustments and strategies enable the effective utilization of coarser powders while maintaining mechanical properties.
- Industry Implications: The insights gleaned from this study have significant implications for industries utilizing PBF-LB/M technology. By leveraging the feedstock cost advantages of coarser powders, manufacturers can achieve substantial cost savings without sacrificing product quality or performance. This not only enhances the economic viability of PBF-LB/M, but also promotes its broader adoption across various sectors.

Moving forward, further research is warranted to explore additional parameters, such as layer thicknesses higher than 60 µm influencing the utilization of PSDs up to 106 µm and higher in PBF-LB/M. Additionally, investigations into the long-term effects of printing with coarser powders, including considerations of part durability, dimensional accuracy, surface roughness, and post-processing requirements, will be crucial for advancing the understanding and maximizing the potential benefits of this approach.

In summary, the findings presented in this study underscore the potential of printing with coarser PSDs as a viable strategy for reducing manufacturing costs and enhancing the competitiveness of PBF-LB/M technology in the additive manufacturing landscape. By bridging the gap between cost-effectiveness and quality, this approach holds promise for unlocking new opportunities and driving innovation in the field of additive manufacturing.

## Figures and Tables

**Table 1:** Ti-6Al-4V industry reference tensile properties [13,32,33].

Powder	Heat Treatment	Layer Thicknessin [µm]	*R_m_* in [N/mm^2^]	*R*_p0.2_ in [N/mm^2^]	*A* in [%]
Xue et al. (horizontal)	800 °C 2 h	60	1029	956	14.3
Xue et al. (vertical)	800 °C 2 h	60	1041	978	16.2
EOS (horizontal)	800 °C 2 h	40	1050	940	14
EOS (vertical)	800 °C 2 h	40	1050	980	15
Nikon SLM (horizontal)	940 °C 4 h	60	987	894	12
Nikon SLM (vertical)	940 °C 4 h	60	991	905	15

**Table 2:** Powder morphology and decile values of the investigated powders.

Powder	SPHT	Symm	w/l	D10 in [µm]	D50 in [µm]	D90 in [µm]
PBF-LB/M_20-53	0.893	0.954	0.896	22.2	33.5	46.8
PBF-EB/M_45-106	0.885	0.926	0.859	51.9	71.2	92.1
Ti64_20-100	0.888	0.943	0.881	25.6	47.3	80.7

**Table 3:** Chemical composition of PBF-LB/M_20-53 and PBF-EB/M_45-106 powders compared to ELI standard [34].

Powder	Ti	Al	V	Fe	C	N
Ti-6Al-4V ELI	balance	5.5–6.5	3.5–4.5	≤0.25	≤0.08	0.03
PBF-LB/M_20-53	balance	6.26	4.02	0.16	0.005	0.014
PBF-EB/M_45-106	balance	6.15	4.0	0.15	0.01	0.02

**Table 4:** Results for apparent density, tapped density, Hausner ratio, and Hall flowability for the investigated powders.

Powder	Apparent Density in [g/cm^3^]	Tapped Density in [g/cm^3^]	Hausner Ratio	Hall Flowability in [s/50 g]	Humidity in [g/cm^3^]
PBF-LB/M_20-53	2.46 ± 0.00	2.68 ± 0.01	1.09	24.67 ± 3.61 ^1^	11.49
Ti64_20-100	2.46 ± 0.01	2.77 ± 0.01	1.13	24.23 ± 0.15	8.13
PBF-EB/M_45-106	2.30 ± 0.01	2.53 ± 0.01	1.10	25.20 ± 0.20	10.29

^1^ no free flow; residual powder in the funnel.

**Table 5:** PBF-LB/M parameter variation and final volume energies and build rates for all investigated powders.

Parameter	PBF-LB/M 20–53 µm	PBF-EB/M 45–106 µm	Ti64 20–100 µm
Build Jobs	2×	1×	3×
Laser Power in [W]	290–310	300–320	280–360
Scanning Speed in [mm/s]	1100–1300	1100–1400	1000–1500
Hatch Distance in [mm]	0.11–0.17	0.08–0.14	0.08–0.14
Final VED in [J/mm^3^]	36.13	47.62	47.62
Final Build Rate in [cm^3^/h]	30.89	24.19	24.19

## Data Availability

The data presented in this study are available on request from the corresponding author. The data are not publicly available due to confidentiality.
